# Miscellany

**Published:** 1906-12

**Authors:** 


### MISCELLANY.

CHARLES S. FOWLER, Chief Examiner, announces that the State Civil Service Commission will hold examinations December 15, 1906, for the following positions: Appraiser of Forest Lands, $600 to $1080 and expenses; Architectural Draughtsman, $15 to $25 a week; Assistant Civil Engineer, $5 to $6 a day; Bridge Designer, $1500 to $1800; Bridge Draughtsman, $1200 to $1500; Chemist, Department of Agriculture, $1200; Civil Engineering Draughtsman, $4 to $5 a day; Fireman, Onondaga County Service, $2 a day; Law Library Assistant, State Library, $600 to $720; Librarian, Department of Labor, $900; Superintendent of Industries, State Prisons, $1500; Tracer, $40 to $75 a month.

The last day for filing applications for these positions is December 10th. Full information and application forms for any of these examinations may be obtained by addressing the Chief Examiner of the Commission at Albany.

